# Whole-genome sequencing and genetic characteristics of representative porcine reproductive and respiratory syndrome virus (PRRSV) isolates in Korea

**DOI:** 10.1186/s12985-022-01790-6

**Published:** 2022-04-11

**Authors:** Seung-Chai Kim, Sung-Hyun Moon, Chang-Gi Jeong, Gyeong-Seo Park, Ji-Young Park, Hye-Young Jeoung, Go-Eun Shin, Mi-Kyeong Ko, Seoung-Hee Kim, Kyoung-Ki Lee, Ho-Seong Cho, Won-Il Kim

**Affiliations:** 1 College of Veterinary Medicine, Jeonbuk National University, 79 Gobong-ro, Iksan, Jeonbuk 54596 Republic of Korea; 2 Animal and Plant Quarantine Agency, 177 Hyeoksin 8-ro, Gimcheon, 39660 Korea

**Keywords:** Genetic diversity, Porcine reproductive and respiratory syndrome virus (PRRSV), Recombination, Phylogenetic analysis, Evolution

## Abstract

**Background:**

Porcine reproductive and respiratory syndrome virus (PRRSV) is a macrophage-tropic arterivirus with extremely high genetic and pathogenic heterogeneity that causes significant economic losses in the swine industry worldwide. PRRSV can be divided into two species [PRRSV1 (European) and PRRSV2 (North American)] and is usually diagnosed and genetically differentiated into several lineages based on the ORF5 gene, which constitutes only 5% of the whole genome. This study was conducted to achieve nonselective amplification and whole-genome sequencing (WGS) based on a simplified sequence-independent, single-primer amplification (SISPA) technique with next-generation sequencing (NGS), and to genetically characterize Korean PRRSV field isolates at the whole genome level.

**Methods:**

The SISPA-NGS method coupled with a bioinformatics pipeline was utilized to retrieve full length PRRSV genomes of 19 representative Korean PRRSV strains by de novo assembly. Phylogenetic analysis, analysis of the insertion and deletion (INDEL) pattern of nonstructural protein 2 (NSP2), and recombination analysis were conducted.

**Results:**

Nineteen complete PRRSV genomes were obtained with a high depth of coverage by the SISPA-NGS method. Korean PRRSV1 belonged to the Korean-specific subtype 1A and vaccine-related subtype 1C lineages, showing no evidence of recombination and divergent genetic heterogeneity with conserved NSP2 deletion patterns. Among Korean PRRSV2 isolates, modified live vaccine (MLV)-related lineage 5 viruses, lineage 1 viruses, and nation-specific Korean lineages (KOR A, B and C) could be identified. The NSP2 deletion pattern of the Korean lineages was consistent with that of the MN-184 strain (lineage 1), which indicates the common ancestor and independent evolution of Korean lineages. Multiple recombination signals were detected from Korean-lineage strains isolated in the 2010s, suggesting natural interlineage recombination between circulating KOR C and MLV strains. Interestingly, the Korean strain GGYC45 was identified as a recombinant KOR C and MLV strain harboring the KOR B ORF5 gene and might be the ancestor of currently circulating KOR B strains. Additionally, two novel lineage 1 recombinants of NADC30-like and NADC34-like viruses were detected.

**Conclusion:**

Genome-wide analysis of Korean PRRSV isolates retrieved by the SISPA-NGS method and de novo assembly, revealed complex evolution and recombination in the field. Therefore, continuous surveillance of PRRSV at the whole genome level should be conducted, and new vaccine strategies for more efficient control of the virus are needed.

**Supplementary Information:**

The online version contains supplementary material available at 10.1186/s12985-022-01790-6.

## Background

Porcine reproductive and respiratory syndrome (PRRS) is one of the most important epidemic diseases affecting the global pig industry and was first identified in the United States in 1987 [1]. The economic impact of the disease in the United States, the pork industry of which accounts for approximately 60% of global pig production, has been estimated at $664 million annually [2, 3]. The causative agent, porcine reproductive and respiratory virus (PRRSV), is an enveloped, single-stranded positive-sense RNA virus with a genome that is approximately 15 kb in length and encodes a 5′ untranslated region (UTR), at least 11 open reading frames (ORFs), a 3′ UTR and a 3′-poly(A) tail [4, 5]. PRRSV is classified into two genotypes, namely, PRRSV1 (EU-type, prototype strain Lelystad virus) and PRRSV2 (NA-type, prototype strain VR-2332 virus), which share only approximately 60% similarity at the nucleotide level [6, 7].

The mutation rate of RNA viruses is high due to the lack of 3′–5′ exonuclease proofreading ability of RNA-dependent RNA polymerase (RdRp), and the calculated rate of nucleotide substitutions in PRRSV is the highest reported for an RNA virus [8, 9]. Among the structural genes of PRRSV, the ORF5 gene encodes the major viral envelope protein GP5, which plays an important role in viral assembly, infectivity and neutralizing antibody induction [10–12]. As ORF5 exhibits high genetic diversity, it is widely used for phylogenetic analysis [13]. Based on the genetic relationships among ORF5 sequences, PRRSV2 is divided into nine lineages, lineage 1 to lineage 9 (L1 to L9), and PRRSV1 is divided into four subtypes, subtype 1 to subtype 4 (sub1 to sub4) [14–16].

In South Korea, PRRSV2 was first detected in the mid-1980s [17], while PRRSV1 was first detected in 2005 and has since spread rapidly [18, 19]. PRRSV1 has been recognized as highly prevalent in Korea and is classified into subgroups A (sub1A), B (sub1B) and C (sub1C) of subtype 1 (Pan-European PRRSV1), with the majority of isolates belonging to sub1A [14, 20]. PRRSV2 has been spreading across the nation for decades, and its genetic diversity has continued to increase through independent evolution, forming unique nation-specific clades designated lineages K and A (LKA), B (LKB) or C (LKC), which are distinct from prevalent global PRRSV strains and commercially modified live vaccine (MLV) strains [14, 21].

Although PRRSV ORF5 has been a very useful tool that has provided insight into the epidemiology of PRRSV, it accounts for only 5% of the whole genome. Thus, whole-genome sequencing (WGS) studies are urgently needed to provide a more complete picture of the virus and obtain identify the remaining 95% of genomic information for prediction of genetic variations [22, 23]. Indeed, based on the whole-genome information of PRRSV strains, studies have identified recombination between two wild-type PRRSV strains [21, 24, 25], between wild-type PRRSV and a MLV strain [26–28] and between MLV strains [29]. RNA recombination has been experimentally suggested to occur in vitro and in vivo in two different strains of PRRSV [30, 31]. It not only results in the generation of novel PRRSV genotypes but is also associated with increases in virulence [32]. Additionally, WGS of PRRSV enabled the analysis of insertion and deletion (INDEL) patterns within the genome. Deletion is often observed in PRRSVs. For example, nonstructural protein 2 (NSP2), which is the largest PRRSV protein, can tolerate amino acid (aa) deletions and foreign gene insertions [33], and it has been reported to be linked to certain patterns, such as a 131-aa discontinuous deletion in NSP2 of MN184- or NADC30-like PRRSV [34], a 100-aa continuous deletion in NSP2 of NADC34-like PRRSV [35], and a 30-aa discontinuous deletion in NSP2 of a highly pathogenic PRRSV strain in China [36].

Since high-throughput next-generation sequencing (NGS) became available in 2005 (Roche 454 FLX platform), NGS technology has become an essential tool for nearly all fields of biological research [37]. However, NGS is not yet in routine use for veterinary testing, and recent studies have attempted to utilize NGS technology for PRRSV WGS based on several different methods and sequencing platforms [23, 38, 39]. Sequence-independent, single-primer amplification (SISPA) is a random priming method that allows the enrichment of the viral genome in only a few steps [40, 41]. The value of the high sensitivity of NGS combined with target-independent genome amplification has been proven via the identification of novel viruses from various samples in previous studies [42–45]. However, to the authors’ knowledge, NGS utilizing the SISPA method (SISPA-NGS) has not been tested or applied in PRRSV.

As the majority of PRRSV genomic sequences submitted to GenBank were contributed by the United States and China [46], the two leading pig-raising countries, the need to identify the prevalent features and genetic variation of unique Korean strains at the whole-genome level has increased [47]. Thus, the purpose of this study was to establish and assess the SISPA-NGS method with the Illumina iSeq 100 platform for PRRSV WGS and to characterize genomic features at the whole-genome level by sequencing archived Korean PRRSV strains.

## Materials and methods

### Cell culture and virus propagation

A total of 19 representative Korean PRRSV isolates that have been most prevalent in the last two decades [48] and two reference strains, namely, VR-2332 (GenBank: AY150164) and JA142 (GenBank: AY424271), were cultured in MARC-145 cells or primary porcine alveolar macrophages (PAMs) in RPMI-1640 medium containing 10% fetal bovine serum and 1% antibiotic–antimycotic at 37 °C in a humid 5% CO_2_ atmosphere. The virus stock was prepared via three consecutive freeze/thaw cycles of the infected cells when approximately 90% of the infected cells showed a cytopathic effect (CPE). The virus-containing cell lysate was clarified by centrifugation at 4000 rpm for 30 min at 4 °C. The clarified samples were filtered through a 0.22-µm-pore-size filter and collected in a sterile container. The collected viruses were kept at − 80 °C until use.

### Virus concentration and RNA preparation

Before applying NGS to the Korean PRRSV strains, we compared PRRSV cDNA preparation procedures using a cell-cultured reference strain (VR-2332) [see Additional file 1]. A total of 8 cDNA preparation methods were tested (designated method 1–method 8). The virus-cultured cell supernatant (method 1 ~ method 4) and concentrated supernatant (method 5–method 8) were used to determine the effect of the PRRSV concentration method. Virus concentration/ultrafiltration was performed with 15 ml of clarified sample by centrifugation at 4000 rpm and 4 °C until all the samples were filtered utilizing a VivaSpin®20 unit with a 300,000 Da molecular weight cutoff (MWCO) (Vivascience) as previously described [49]. Viral RNA was extracted using a QIAamp Viral RNA Mini Kit (Qiagen) according to the manufacturer’s instructions. The extracted RNA was treated with a DNase Max Kit (Qiagen) and purified with Agencourt RNA Clean XP beads (Beckman Coulter) following the manufacturer’s instructions. Purified RNA was examined with a NanoDrop spectrophotometer and a Qubit 2.0 fluorometer using a Qubit RNA BR Assay Kit. Real-time reverse transcription-polymerase chain reaction (RT–qPCR) was performed for the quantification of viral RNA using the Prime-Q PCV2 PRRSV Detection Kit (Genet Bio, Daejeon, South Korea). The prepared RNAs were stored at − 80 °C until use.

### cDNA preparation and next-generation sequencing

Different combinations of simple random hexamer cDNA synthesis (RH), RNA fragmentation (RF), and SISPA techniques for generating double-stranded (ds) cDNA were tested: methods 1 and 5, RH; methods 2 and 6, RF + RH; methods 3 and 7, SISPA; and methods 4 and 8; RF + SISPA [see Additional file 1] [38, 40, 43, 50]. For RF, 100 ng of the prepared RNA was fragmented using a NEBNext Magnesium RNA Fragmentation Module (NEB) with a 2 min incubation time, and fragmented RNA was purified using the GeneJet RNA Cleanup and Concentration Micro Kit following the manufacturer’s instructions. A 100-ng sample of fragmented or nonfragmented RNA was used for conduct reverse transcription (SuperScript IV First-Stand Synthesis System, Invitrogen) with random hexamers provided in the kit (methods 1, 2, 5, and 6) or with the SISPA primer (K-6 N) (methods 3, 4, 7, and 8). The synthesized first-strand cDNA was subsequently treated with 1 µl of RNase H for 30 min at 37 °C. For the RH and RF + RH methods, 21 µl of first-strand cDNA was incubated at room temperature (RT) for 30 min in the presence of 1 µl of 10 mM dNTPs, 1 µl of Klenow fragment, 1 µl of *E. coli* DNA ligase (NEB), 5 µl of 10 × *E. coli* ligation buffer (NEB) and 21 µl of DEPC-treated water (final volume 50 µl). For the SISPA and RF + SISPA methods, 21 µl of first-strand cDNA, 1 µl of 100 pmol of SISPA primer (K-6N), 1 µl of 10 mM dNTPs, 1 µl of Klenow fragment, 5 µl of 10 × Klenow reaction buffer (NEB) and 21 µl of DEPC-treated water (final volume 50 µl) were mixed and incubated at RT for 30 min. After conversion to ds cDNA by the Klenow reaction, the products were purified using Agencourt AMPure XP beads (Beckman Coulter). For methods including SISPA, 5 µl of the ds cDNA was used as template for PCR amplification in a final reaction volume of 50 µl, which contained 1 × Platinum SuperFi II Green PCR Master Mix (Thermo Fisher) and 10 µM SISPA primer (K). PCR cycling was performed as follows: 98 °C for 30 s, followed by 35 cycles of 98 °C for 10 s, 60 °C for 10 s, and 72 °C for 30 s, with a final extension at 72 °C for 5 min. The PCR products were purified with Agencourt AMPure XP beads (Beckman Coulter). The final ds cDNA products were quantified using a Qubit dsDNA HS Assay Kit (Invitrogen).

One hundred nanograms of the prepared ds-cDNA and the Nextera DNA Flex Library Prep Kit were used to generate multiplexed paired-end sequencing libraries according to the manufacturer’s instructions. The prepared sequencing libraries were analyzed with a High Sensitivity DNA Chip on a Bioanalyzer (Agilent Technologies). A barcoded multiplexed library pooled with 3% PhiX (positive control) was sequenced on the Illumina iSeq 100 platform (2 × 150 bp).

### Bioinformatics

The adaptor and index sequences of the reads were trimmed, and low-quality sequences were filtered out using AfterQC version 0.9.6 [51]. The filtered reads were mapped to the VR-2332 reference genome (AY150564) and the host genome (AQIB01) using Minimap2 [52] version 2.17 with the following command modifications of the default parameters: -k 7 for virus and -k 14 for host. Read coverage was calculated using QualiMap version 2.2.1 [53]. Host-unmapped reads were extracted using SAMtools [54] and subsequently used for de novo assembly by SPAdes version 3.14.1 with the -meta command [55]. The generated contigs were subjected to BLAST analysis to identify the longest PRRSV-matching contig. The longest contigs that were close to the genome size of VR-2332 (15,451 bp) were used for quality assessment of the genome assembly with Quast version 5.0.2 [56]. Reference sequences and assembled contigs were aligned using Lasergene software (DNASTAR Inc.) to assess the ability to sequence the 5′ and 3′UTRs.

### WGS of Korean PRRSV isolates

Subsequently, a total of 20 PRRSV strains that were isolated and archived in our laboratory were sequenced via the established SISPA-NGS procedure (method_7) with a maximum of 6 samples per sequencing batch. Among these strains, the WGS of JA142 (AY424271), CBNU0495 (KY434183) and D40 (KY434184) was performed as a resequencing analysis of the known genome to confirm the fidelity of the established procedure, and the other Korean PRRSV strains were sequenced for novel genome assembly. For data analysis, filtering of the raw data and de novo assembly were conducted as described above. Newly obtained Korean PRRSV whole genome sequences were submitted to GenBank under accession numbers MW847781 and MZ287313–MZ287330. To determine the cutoff Cq value for successful WGS, the Prime-Q PCV2 PRRSV Detection Kit (GeNet Bio Inc., Daejun, Korea) was used to extract RNAs according to the manufacturer’s instructions.

### Phylogenetic analysis

A total of 69 representative PRRSV strains were obtained from the GenBank database, including all the Korean strains that were available in the database as of November 2020 [see Additional file 2]. All the sequences were aligned using Clustal Omega, including the 19 newly obtained whole-genome sequences from this study [57]. Phylogenetic trees of the complete genome and ORF5 were constructed by using RAxML-NG [58] with 1,000 bootstrap replicates and the GTRGAMMA nucleotide substitution model. The constructed trees were visualized and edited using FigTree software [59].

### NSP2 polymorphic pattern analysis

To identify the various INDEL patterns of NSP2, all the complete sequences described above were split and aligned with the Lelystad virus (PRRSV1) or VR-2332 (PRRSV2) as a reference using Clustal Omega. The alignment was split to obtain NSP2 sequences based on the reference annotation and realigned based on aa sequences by using Clustal Omega. The aligned aa sequences were then converted to a nucleotide alignment with PAL2NAL software to obtain an accurate alignment as previously described [46, 60]. The INDEL patterns of NSP2 at the aa level were visualized using the NCBI Multiple Sequence Alignment (MSA) tool.

### SimPlot and recombination analysis

To detect recombination events among the Korean strains, nine lineage representative strains (Amervac, KNU07, CBNU0495, RespPRRS_MLV, NADC30, NADC34, K07-2273, JB15-N-PJ10-GN and KU-N1606) were selected based on time inferences and previous reports of the epidemiological status of PRRSV in Korea [14, 18–21, 47, 61, 62]. After the alignment of whole-genome sequences, the representative strains listed above were queried as recombinant parental strains and analyzed with SimPlot version 3.5.1 [63] with a window size of 500 bp and a step size of 20 bp to identify potential recombination events and breakpoints. Subsequently, the sequences were analyzed using RDP v4.9.6 [64], and recombination events were indicated when seven methods reported positive recombination signals (RDP, GENECONV, BootScan, Maxchi, Chimaera, SiScan, and 3Seq). The final recombination events were determined after taking into consideration both the SimPlot and RDP results.

## Results

### Comparison of cDNA preparation methods

All the methods resulted in nearly 100% mapping to the reference, but the methods that included the SISPA protocol showed 100% coverage with greater depth than the other methods and generated fewer low-quality reads than the methods using random hexamers for ds cDNA construction [see Additional file 3]. Although complete or near-complete virus genome sequences could be obtained by de novo assembly by all the methods [see Additional file 4], only assembly via method_7 (virus concentration + SISPA) allowed the full sequences of the 5′ and 3′ UTRs of the virus to be retrieved [see Additional file 5].

### PRRSV WGS with the SISPA-NGS method

**Fig. 1:**
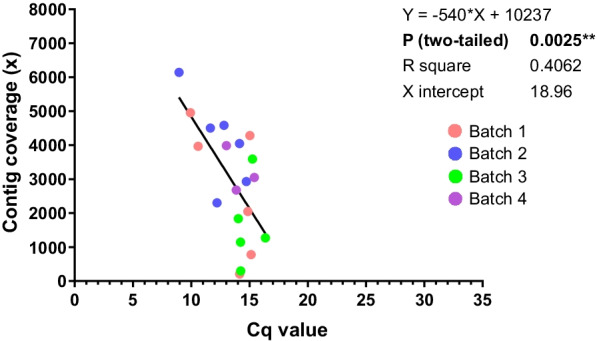
Correlation between contig coverage and virus Cq value for de novo assembly. Different colors indicate each batch of sequencing conducted on the iSeq 100 platform

To obtain the whole-genome sequences of all the Korean PRRSV strains and reference strains, the ds-cDNAs of all the virus stocks were constructed using the SISPA-NGS method (method_7) and sequenced on the iSeq100 platform (Table 1). The assembled sequences of the JA142, D40 and CBNU0495 strains, which were used as resequencing controls, showed highly consistent nucleotide identity with the corresponding reference sequence (99.87% to 99.97%) [see Additional file 6] (Table S4). The whole-genome sequences of all the viral stocks were successfully assembled without any gaps, allowing the recovery of complete or near-complete genome sequences, while the average contig read depth differed according to the Cq values of the stocks (Table 1). The read depth of the PRRSV genomic sequences decreased as the Cq value decreased, and the approximate cutoff Cq value for whole-genome assembly was 18.96 (Fig. 1).

**Table 1:** De novo assembly of PRRSV strains in this study

Strain	Type	Year isolated	Cq value	Total reads	Final contig length (bp)	Depth of coverage ( ×)	ORF5 lineage^a^
JA-142	PRRSV2	2003	9.93	774,372	15,386	4954	L8
K07-2273	PRRSV2	2007	14.16	946,690	14,904	208	LKC
K08-1054	PRRSV2	2008	10.59	862,281	15,382	3965	L5
CBJE19	PRRSV2	2010	14.23	548,750	14,989	1145	LKA
GGYC45	PRRSV2	2010	16.37	551,128	14,977	1273	LKB
D40	PRRSV1	2011	15.14	775,059	14,992	779	sub1A
CNCY42	PRRSV2	2011	11.65	917,738	15,385	4504	L5
GBGJ22	PRRSV2	2011	15.26	599,160	15,003	3591	LKA
JB15-N-PJ4-GN	PRRSV2	2015	15.04	1,093,345	15,384	4284	L5
JB15-N-M8-GN	PRRSV2	2015	8.95	708,664	14,962	5962	L5
JB15-N-PJ10-GN	PRRSV2	2015	12.81	833,933	14,989	4585	LKB
JB15-N-P31-GB	PRRSV2	2015	14.14	964,440	14,970	4048	L1
JB15-N-PJ45-GN	PRRSV2	2015	12.22	952,476	14,983	2305	LKC
JB15-N-PJ73-GN	PRRSV2	2015	14.73	861,340	14,975	2928	L1
JB15-E-M17-JB	PRRSV1	2015	14.04	748,606	15,111	1835	sub1C
JB15-E-P47-GB	PRRSV1	2015	14.24	619,588	14,965	298	sub1A
CBNU0495	PRRSV1	2016	14.86	806,549	14,942	2046	sub1A
JBNU-19-E01	PRRSV1	2019	15.41	1,301,645	14,993	3052	sub1A
JBNU-19-N01	PRRSV2	2019	13.02	1,498,543	14,952	3985	L1
JBNU-20-N01	PRRSV2	2020	13.86	1,335,257	14,988	2681	L1

^a^The classified ORF5 lineage based on previous studies[16, 48]

### Phylogenetics and NSP2 INDEL patterns of Korean PRRSV1

**Fig. 2:**
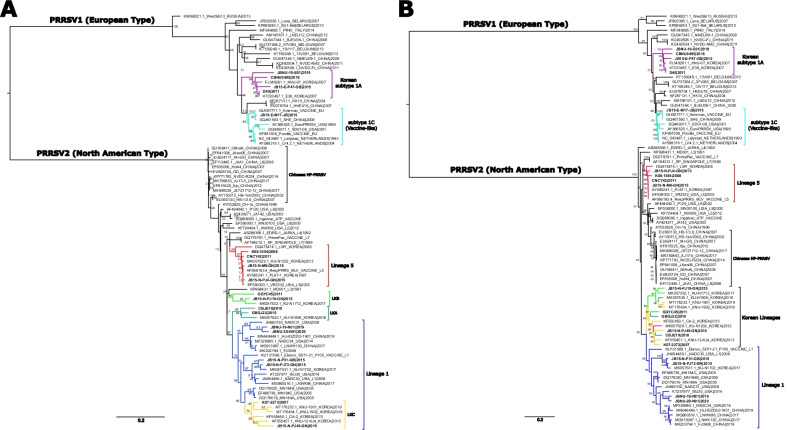
Midpoint-rooted phylogenetic tree of **a** ORF5 and **b** the whole-genome sequences of Korean isolates and representative reference strains. Korean isolates newly sequenced in this study are indicated in bold. Lineages of PRRSV1 and PRRSV2 are color-coded and include lineages 1 (blue), 5 (red), KOR A (teal), KOR B (lime), KOR C (orange), sub1A (purple), and sub1C (aqua). Trees were generated by RAxML-NG with 1,000 bootstrap replicates using the GTRGAMMA nucleotide substitution model

**Fig. 3:**
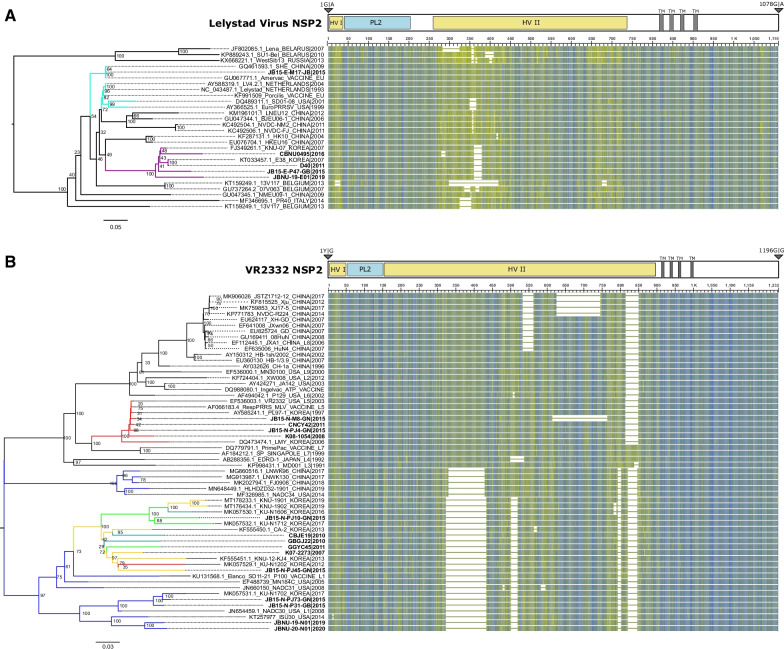
Schematic diagram of multiple alignments of NSP2 amino acid sequences relative to **a** the Lelystad virus (PRRSV1) and **b** VR-2332 (PRRSV2). The illustration on the top represents the organization of the PRRSV NSP2 protein, featuring a putative cysteine domain (PL2) between two hypervariable (HV) regions. The trees on the left were generated and color-coded based on NSP2 nucleotide sequences, as indicated in Fig. 2. Multiple sequence alignments (MSAs) were generated by using the NCBI MSA tool version 1.18, with coloration based on the BLOSUM62 matrix. Detailed MSAs of the PRRSV1 and PRRSV2 isolates are illustrated in Additional files 7 and 8, respectively

Phylogenetic trees were constructed based on the ORF5, whole-genome and NSP2 nucleotide sequences of the Korean PRRSV strains to identify genetic characteristics at the level of each gene or genome (Fig. 2). Among seven Korean PRRSV1 isolates, six isolates could be grouped into PRRSV1 sub1A, which formed a unique branch, separate from those of PRRSV1 strains from other countries, at the ORF5 and whole-genome levels. One Korean PRRSV1 isolate was close to strain SHE and the Amervac vaccine strain at the ORF5 and whole-genome levels and was included in the PRRSV1 sub1C group (Fig. 2). At the NSP2 level, Korean PRRSV1 showed the same phylogenetic pattern observed at the ORF5 and whole-genome levels. However, NSP2 INDEL pattern analysis revealed that all the Korean PRRSV1 sub1A isolates showed a unique 19-aa deletion (aa positions 361–379 in the Lelystad virus), and one strain, CBNU0495, showed an additional 11-aa deletion (aa positions 280–290) (Fig. 3a and Additional file 7).

### Phylogenetic analysis and NSP2 INDEL patterns of Korean PRRSV2

With regard to PRRSV2, all the Korean PRRSV2 isolates could be divided by ORF5 lineage classification into lineage 5, lineage 1 and Korean-specific clusters (LKA, LKB, and LKC) (Fig. 2a). The Korean strains classified into lineage 5 were related to the RespPRRS_MLV strain at the ORF5 and NSP2 levels and at the whole-genome level, indicating that they are MLV-like viruses. Two strains from lineage 5 showed different patterns from the other MLV-like strains. Strain JB15-N-M8 showed a unique 150-aa deletion (aa position; 615–764 in VR-2332) in the hypervariable (HV) region of NSP2. Strain KU-N1202, which was previously reported as a recombinant strain [21], exhibited an ORF5 phylogeny close to that of MLV strains. However, at the whole-genome and NSP2 levels, the strain was clustered into the Korean lineage group (Figs. 2, 3b and Additional file 8).

The Korean strains within lineage 1 could be divided into two groups at the ORF5 and whole-genome levels by phylogenetic analysis; one group (JB15-NP31-GB, JB15-N-PJ73-GN, and KU-N1702 strain) was close to the highly pathogenic NADC30 strain, and the other group (JBNU-19-N01 and JBNU-20-N01 strain) was relatively close to the NADC31 or NADC34 strain (Fig. 2). However, all the Korean lineage 1 strains in both groups were close to NADC30 in the NSP2 phylogenetic analysis. The NSP2 aa structures of both groups showed the same deletion pattern of 111-aa (aa positions 323–433 in VR-2332), 1-aa (aa position 485), and 19-aa (aa positions 501–519) deletions (“111 + 1 + 19”) in the HV region of NSP2, which was consistent with the findings for the NADC30 strain (Fig. 3b and Additional file 8).

The Korean strains that could be clearly classified into three distinct Korean-specific clusters (LKA, B and C) by ORF5 phylogenetic analysis were merged into one large branch with two subbranches by whole-genome and NSP2 phylogenetic analysis, indicating that they shared a potential common ancestor or showed recombination (Figs. 2, 3b). Interestingly, whole-genome phylogenetic analysis demonstrated that the strains that were isolated before 2015 formed one subbranch, and those that were isolated after 2015 formed the other subbranch (Fig. 2b). All the Korean lineage strains shared a common “111 + 1 + 19” deletion pattern in the HV region, which was exactly the same pattern observed for the NADC30 strain or K07-2273 strain (Fig. 3b and Additional file 8).

### Recombination analysis of Korean PRRSV2

**Fig. 4:**
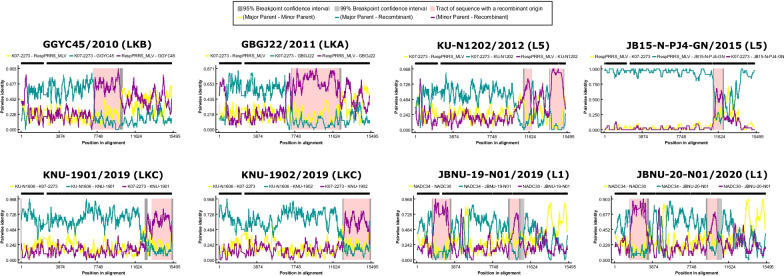
Recombination detection in Korean isolates. The x-axis indicates the genomic position, and the y-axis indicates the pairwise identity between the major parent and minor parent (yellow), between the major parent and the recombinant strain (green), or between the minor parent and the recombinant strain (purple). The range of recombination is shaded in red

As a few Korean PRRSV2 isolates showed the possibility of recombination in the ORF5, whole-genome and NSP2 phylogenetic analyses, SimPlot software was utilized to determine potential recombination events and breakpoints by querying the potential parental strains based on the corresponding time inferences and impacts [see Additional file 9]. Strains RespPRRS_MLV (L5), NADC30 (L1), NADC34 (L1), KU-N1606 (LKA), 2015-N-PJ10-GN (LKB) and K07-2273 (LKC) were selected as potential parents of other Korean strains. Subsequent genetic recombination analysis performed with seven methods in RDP4 software indicated recombination signals in 8 Korean PRRSV strains (Table 2, Fig. 4, and Additional file 10).

**Table 2:** Recombination events identified by SimPlot and RDP 4 software

Recombinant	Major parent	Minor parent	Breakpoint positions^a^ (nt)			References
			Beginning	End	Region	
GGYC45/2010	K07-2273/2007	RespPRRS_MLV/Vaccine	7012	9613	NSP8—NSP10	This study
GBGJ22/2011	K07-2273/2007	RespPRRS_MLV/Vaccine	7038	11,845	NSP8—NSP12	This study
KU-N1202/2012	K07-2273/2007	RespPRRS_MLV/Vaccine	10,772	11,408	NSP11	[21]
			13,365	14,708	ORF5—ORF6	
JB15-N-PJ4-GN/2015	RespPRRS_MLV/Vaccine	K07-2273/2007	11,164	12,029	NSP11—NSP12	This study
KNU-1901/2019	KU-N1606/2016	K07-2273/2007	12,303	14,879	ORF3—ORF7	[47]
KNU-1902/2019	KU-N1606/2016	K07-2273/2007	12,303	14,879	ORF3—ORF7	
JBNU-19-N01/2019	NADC34/2014	NADC30/2008	1651	2948	NSP2	This study
			9060	10,003	NSP10	
JBNU-20-N01/2020	NADC34/2014	NADC30/2008	1651	2948	NSP2	This study
			9060	10,003	NSP10	

^a^Recombination breakpoints based on the nucleotide position of the identified recombinant strain

The K07-2273 strain (LKC, isolated in 2007) was the major parent of all the recombinant strains isolated before 2016. All of these recombinants showed the vaccine strain RespPRRS_MLV as the minor parent, with recombination hotspots ranging from NSP8 and NSP12. Additionally, these recombinant strains possessed different ORF5 lineage phylogenies (GGYC45; LKB, GBGJ22; LKA, and KU-N1202 and JB15-N-PJ4-GN; L5) from their major parent strain, K07-2273 (LKC) (Fig. 2), indicating that the KLC lineage might have been responsible for the emergence of nation-specific Korean lineages through recombination with the MLV strain after vaccine introduction in Korea in the early 2010s.

After 2016, since the LKB and LKC strains emerged, the KNU-1901 and KNU-1902 strains showed recombination signals with KU-N1606 (LKA) as the major strain and K07-2273 (LKC) as the minor strain, with the recombination regions ranging from ORF3 to ORF7. Among lineage 1 strains, the JBNU-19-N01 and JBNU-20-N01 strains showed recombination signals with NADC30 as the major strain and NADC34 as the minor strain, with recombination regions of NSP2 and NSP10 (Table 2). No potential recombination event or breakpoint was detected in the PRRSV1 isolates.

## Discussion

The epidemiology of PRRSV has been investigated largely by sequencing the ORF5 gene and classifying virus lineages based on ORF5 phylogenetic analysis since the ORF5 lineage classification system was established [15, 16]. However, due to the error-prone nature of PRRSV RdRp and frequent recombination among field strains or vaccine strains, an urgent need for WGS and genome-based analysis has been demonstrated recently. In Korea, ORF5 sequencing has been used in most diagnostic laboratories and has identified PRRSV outbreaks and the emergence of Korean nation-specific lineages [14, 18–20, 62, 65]. However, the origin and genetic characteristics of these novel lineages remain unclear due to the limited available whole-genome information, as only 12 PRRSV whole-genome sequences are available in the GenBank database. Furthermore, recent reports of recombinant strains in Korea indicate that ORF5 sequencing is no longer sufficient for the complete identification or classification of PRRSV isolates [21, 47, 61, 66]. Thus, the purposes of this study were to establish a WGS method for retrieving PRRSV whole-genome sequences and to analyze the PRRSV genetic diversity in Korea at the whole-genome level.

WGS provides an unsurpassed level of genetic information. In this study, we compared cDNA preparation methods using random hexamers with or without RNA fragmentation and SISPA with or without RNA fragmentation [see Additional file 1]. SISPA is a random priming method that allows the enrichment of a viral genome in only a few steps, which is very useful for obtaining genome sequences from RNA viruses or complex clinical samples, as no prior sequence information is needed [43]. As expected, more virus reads could be generated from samples using SISPA than by using non-SISPA methods [see Additional file 3], resulting in the retrieval of complete genome sequences with a higher depth of coverage [see Additional files 4 and 5]. Moreover, we applied a 300,000 Da MWCO membrane to concentrate PRRSV particles from the cell-cultured supernatant as previously described [49], resulting in a higher initial virus titer, shown as a lower Cq value [see Additional file 3]. Such virus filtration or concentration protocols are common methods for capturing and sequencing unknown virus pools from samples with large volumes, such as ocean water samples [67, 68]. With the advantages mentioned above, the established SISPA-NGS method coupled with virus concentration in this study enabled the successful WGS of cell-cultured PRRSV isolates without prior sequence information (Table 1 and Additional file 6). However, the detection limit based on the Cq value (18.96) was lower than that indicated in a previous report that used the MiSeq platform (20.6 ~ 23.6) [38] (Fig. 1). This might have been due to the reduced data output of the iSeq100 platform, which allows smaller batch sizes than higher-throughput sequencers, such as the MiSeq system [69], resulting in a less sufficient depth of coverage for de novo assembly. It will be necessary to test the established NGS method with clinical samples and other sequencers in the future.

The Korean PRRSV strains newly sequenced in this study were classified into distinct lineages by the ORF5 lineage classification system as previously reported in Korea [14, 48]: subtype 1A, subtype 1C, lineage 1, lineage 5, lineage KOR A (LKA), KOR B (LKB), and KOR C (LKC) (Fig. 2a). With regard to PRRSV1 (EU type), phylogenetic analysis of the whole genome showed that Korean PRRSV subtype 1A viruses are highly divergent from viruses identified from other countries and share a low level of identity with each other (Fig. 2b), which is consistent with previous studies [18, 19]. As PRRSV1 continued to become increasingly prevalent and to impose an enormous economic burden in Korea, two PRRSV1 MLVs [Unistrain PRRS (Amervac) and Porcilis PRRS)] were introduced in Korea in 2014 [66]. A vaccine-like strain (subtype 1C) isolated in this study (strain JB15-E-M17-JB) shares > 99% nucleotide homology with the Unistrain PRRS or SHE strain at the whole-genome level (Fig. 2b). PRRSV1 subtype 1A strains isolated in Korea after 2015 (JB15-E-P47-GB, CBNU0495, and JBNU-19-E01 strains) are highly divergent from those isolated before 2015 (KNU-07, E38, and D40 strains) at the whole-genome level (Fig. 2b). As NSP2 INDEL pattern analysis indicated a consistent 19-aa deletion among PRRSV1 subtype 1A strains (Fig. 3a and Additional file 7) and no recombination signal was detected among PRRSV1 strains, it is suspected that increased independent evolution of Korean PRRSV1 subtype 1A is ongoing in the field. Indeed, a recent study in Korea revealed that PRRSV1 MLV vaccination increased viral genetic heterogeneity and the emergence of new glycosylation sites among Korean subtype 1A viruses when comparisons were conducted before and after vaccine adoption in the field [66]. Continuous surveillance of the dynamics of PRRSV1 evolution in Korea should be further performed at the whole-genome level.

With regard to PRRSV2, vaccine variants of Ingelvac MLV (RespPRRS_MLV) with high nucleotide homology (> 95%) and a consistent NSP2 INDEL pattern were confirmed, except that the JB15-N-M8 strain had a unique 150-aa deletion in the HV region (Figs. 2b, 3b and Additional file 8). In lineage 1, NADC30-like strains (sublineage 1.8) that have emerged since 2015 in Korea [14] and non-NADC30-like strains that were closely related to the ISU30 strain at the whole-genome level were detected based on whole-genome phylogenetic analysis (Fig. 2b). These non-NADC30-like viruses (strains JBNU-19-N01 and JBNU-20-N01) were first isolated in Korea in 2017 [48] and were classified into sublineage 1.6 (close to strain NADC31) based on ORF5 (Fig. 2a). However, these viruses were confirmed to be recombinants of NADC30 and NADC34 through RDP and SimPlot analyses (Table 2 and Additional file 9). The NADC34-like (or RFLP 1–7-4) lineage, which emerged in the United States in 2014, is a highly pathogenic cluster of lineage 1 that causes dramatic abortion ‘storms’ in sow herds and high mortality among piglets [35]. It was subsequently detected in China beginning in 2017 [70], and some Chinese NADC34-like strains were confirmed to be recombinants of NADC34 as the major parent and NADC30 or ISU30 as the minor parent, with recombination in the NSP2 or ORF4-5 region [71]. Likewise, the lineage 1 recombinants identified in this study showed recombination involving NADC34 as the major parent and NADC30 as the minor parent, with recombination signals in the NSP2 and NSP10 regions (Table 2 and Additional file 9). Although it is not clear whether the observed recombination occurred among Korean field isolates or the recombinant strains were initially imported from outside of the country, lineage 1 viruses have been confirmed to be spreading extensively in Korea since 2017 [48]. Thus, the clinical manifestations and continual surveillance of these lineage 1 recombinants should be further assessed and monitored.

**Fig. 5:**
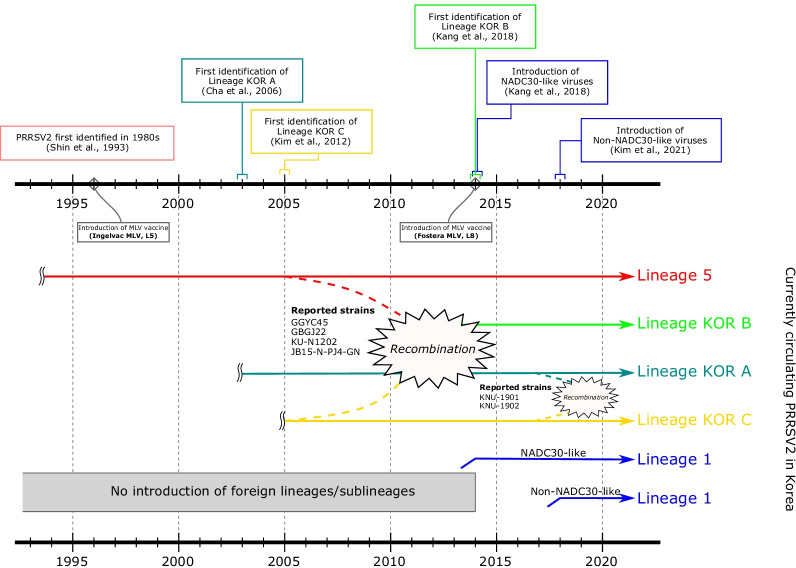
Schematic diagrams of PRRSV2 epidemiology in Korean pig herds. The top illustration shows the timeline of PRRSV2 epidemiology in Korea based on previous reports. The bottom illustration indicates the spreading of each lineage and recombination event

Recombination is a pervasive phenomenon among PRRSV isolates and is an important strategy for the generation of viral genetic diversity as a result of increased virulence and/or the generation of novel genotypes [30, 32, 72–74]. As variants of PRRSV2, Korean lineages (LKA, B and C) have been found to be prevalent in Korean swine herds, but the origin of these nation-specific lineages is currently unknown [14, 48]. Considering the epidemiology of PRRSV2 in Korea, it is suspected that genomic recombination between the LKC and Ingelvac MLV (RespPRRS_MLV) strains might have been involved in the generation of LKB (Fig. 5). Following the first identification of PRRSV2 in Korea in the 1980s [17], the Ingelvac MLV was commercialized in Korea in 1996 [75]. LKA and LKC have been isolated from Korean swine herds since 2003 and 2005, respectively [62, 75]. A study that investigated ORF5 sequences collected from 2003 to 2016 indicated that LKB was first detected in samples collected since 2014 [14]. In this study, multiple recombination signals were detected among Korean lineages, with K07-2273 (LKC) as the major parent and Ingelvac MLV as the minor parent of the strains isolated in the early 2010s (Table 2 and Fig. 4). The analysis of NSP2 INDEL patterns suggested that the Korean lineages originated from the same parents, as identical discontinuous 131-aa deletion patterns were identified in the HV region of NSP2 (Fig. 3b and Additional file 8), which is a well-established pattern in NADC30-like or MN184-like viruses [34, 76]. Among the recombinants identified among the isolates from the early 2010s, the GGYC45 strain (isolated in 2010) is suspected to be the ancestor of current LKB strains, as it is the earliest strain that possesses the ORF5 gene of LKB, since LKB strains emerged in 2014. Given that Korean PRRSV2 evolved independently with genetic heterogeneity in antigenic regions of structural proteins [61], recombination between the Korean-specific lineage (LKC) and the MLV strain in NSP regions, together with evolution in the structural protein regions that resulted in the LKB-like ORF5 gene, might have induced the emergence of a novel sublineage. Although more evidence should be collected and analyzed, it is well known that the extensive use of live-attenuated vaccines contributes to the increased PRRSV genetic diversity in the field [77, 78], and several studies have reported PRRSV2 recombinants involving MLVs [79, 80]. Since multiple lineages of PRRSV2, including MLV variants, Korean lineages and imported lineage 1 viruses are circulating in Korea [48], there is a potential risk of recombination between different lineages. Further research on PRRSV evolution followed by continuous surveillance at the whole-genome level in Korea is needed.

## Conclusions

In summary, we established NGS using the SISPA protocol for successful WGS of PRRSV field isolates. Genomic sequence analysis revealed continuous independent evolution of PRRSV1 and evidence of multiple recombination events between PRRSV2 MLV variants and Korean lineages that are prevalent in Korea. Additionally, potential recombinant NADC30-like and NADC34-like strains could be detected in this study. As Korea is an isolated country with very tight borders, the independent evolutionary dynamics observed in Korea could contribute to the general knowledge of PRRSV evolution. Our study highlights the importance of continued monitoring of PRRSV and new vaccine strategies for more efficient control of the virus.

## Supplementary Information

**Additional file 1**. Workflow of the comparison of eight methods of cDNA preparation for NGS.

**Additional file 2**. Representative PRRSV strains used in this study.

**Additional file 3**. FASTQ quality control and mapping assessment of eight optimized cDNA preparation methods.

**Additional file 4**. De novo assembly assessment by QUAST with contigs aligned to the reference genome.

**Additional file 5**. Multiple sequence alignment of the reference genome and contigs generated from eight methods of cDNA preparation showing the 5′ and 3′UTRs.

**Additional file 6**. BLAST results of newly assembled PRRSV whole-genome sequences.

**Additional file 7**. Multiple sequence alignment of PRRSV1 NSP2.

**Additional file 8**. Multiple sequence alignment of PRRSV2 NSP2, with the “111 + 1 + 19” deletions indicated with black dashed-line boxes.

**Additional file 9**. SimPlot analysis querying potential major parent strains against Korean PRRSV isolates.

**Additional file 10**. *P value* of seven methods (RDP, GENECONV, BootScan, Maxchi, Chimaera, SiScan, and 3Seq) applied in RDP4 software.

## Data Availability

All data generated or analyzed during this study are included in this published article (and its supplementary information files).

## Abbreviations

PRRSV: Porcine reproductive and respiratory syndrome virus
SISPA: Sequence-independent, single-primer amplification
NGS: Next-generation sequencing
UTR: Untranslated region
RdRp: RNA-dependent RNA polymerase
ORF: Open reading frame
NSP: Nonstructural protein
INDEL: Insertion and deletion
MLV: Modified live vaccine
WGS: Whole-genome sequencing
PAM: Porcine alveolar macrophage
CPE: Cytopathic effect
RH: Random hexamer
RF: RNA fragmentation
RT: Room temperature
