# Supplementary figures and images for: Whole-genome sequencing and genetic characteristics of representative porcine reproductive and respiratory syndrome virus (PRRSV) isolates in Korea
Source: Virol J. 2022 Apr 11;19:66. doi: 10.1186/s12985-022-01790-6 (PMC8996673; doi:10.1186/s12985-022-01790-6)

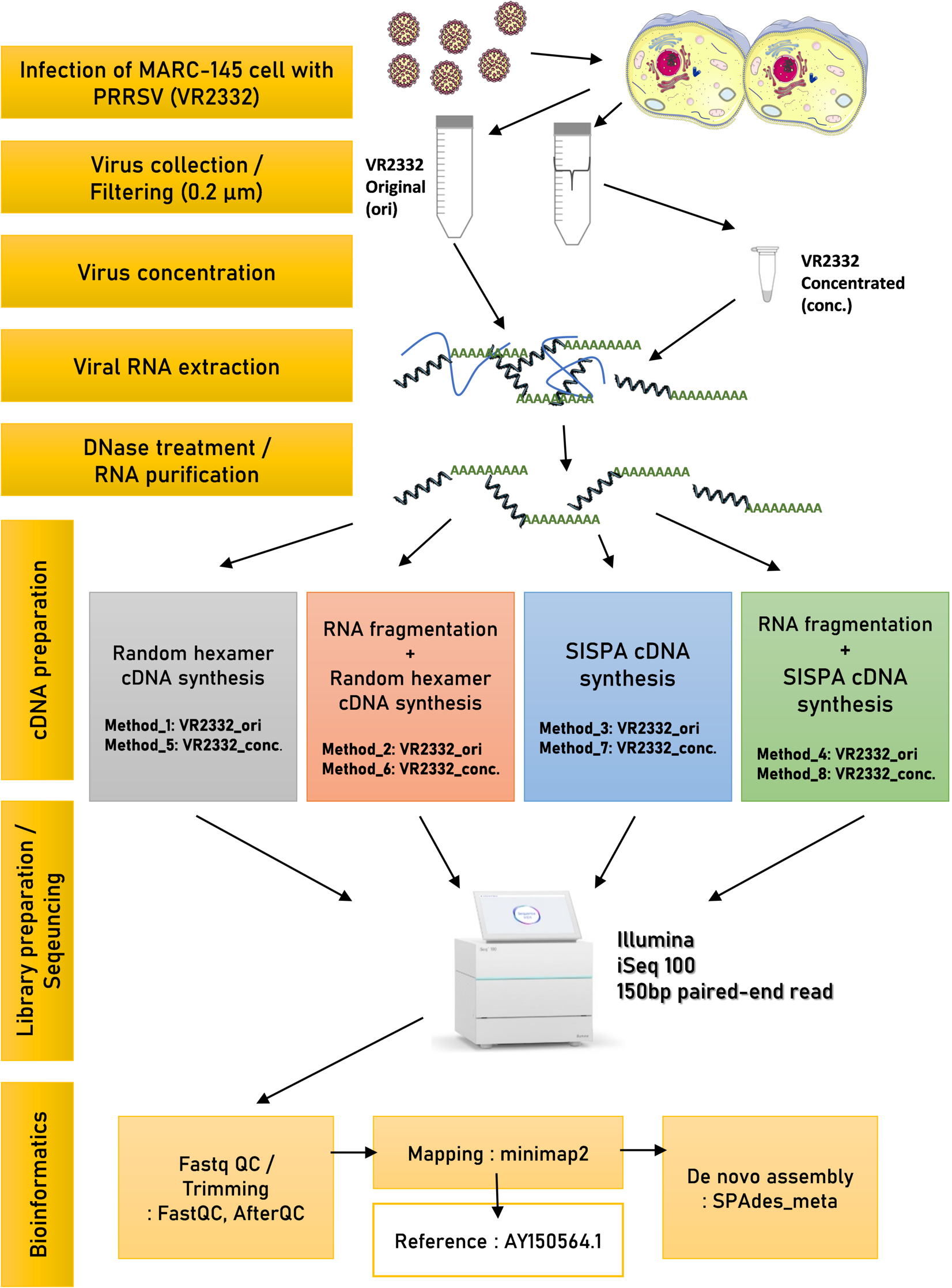

Supplement: Supplementary file 2 — Additional file 2. Representative PRRSV strains used in this study. [file 12985_2022_1790_MOESM2_ESM.pdf]

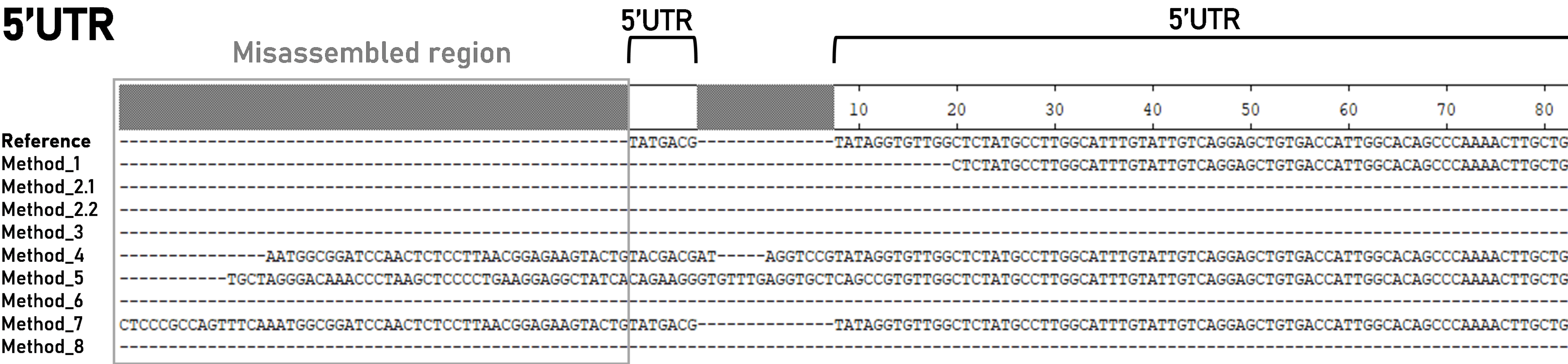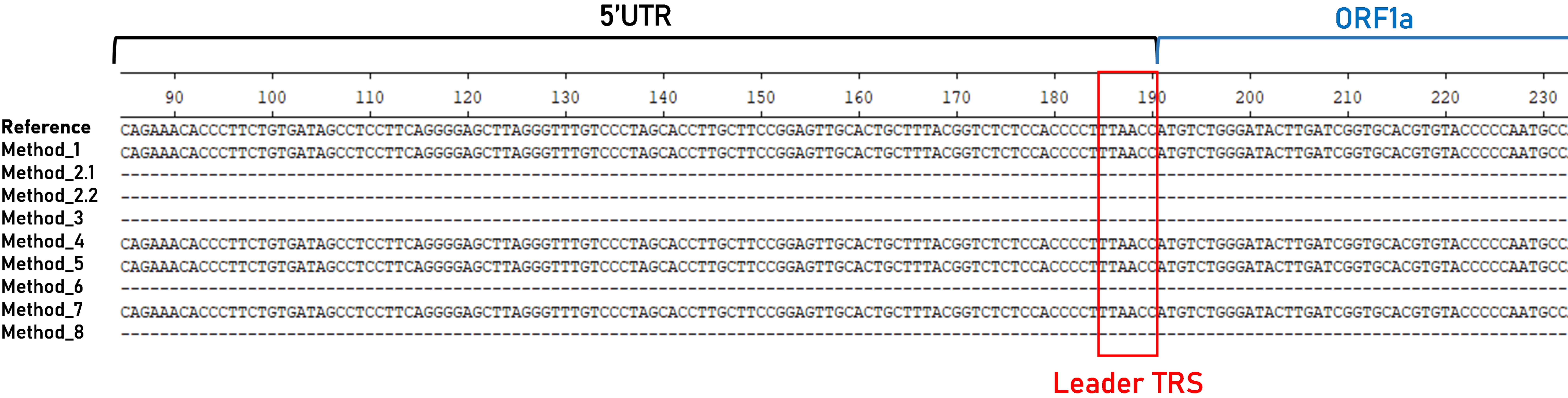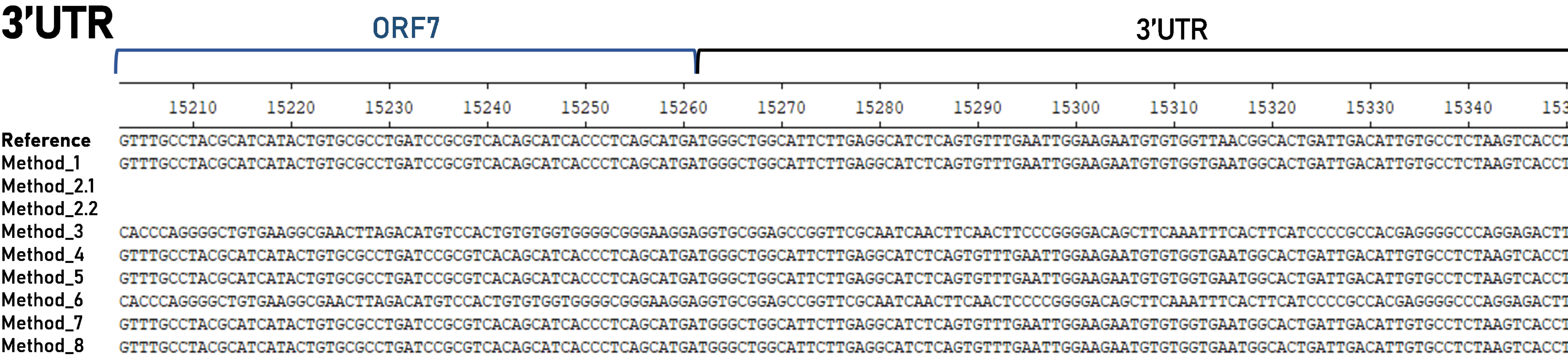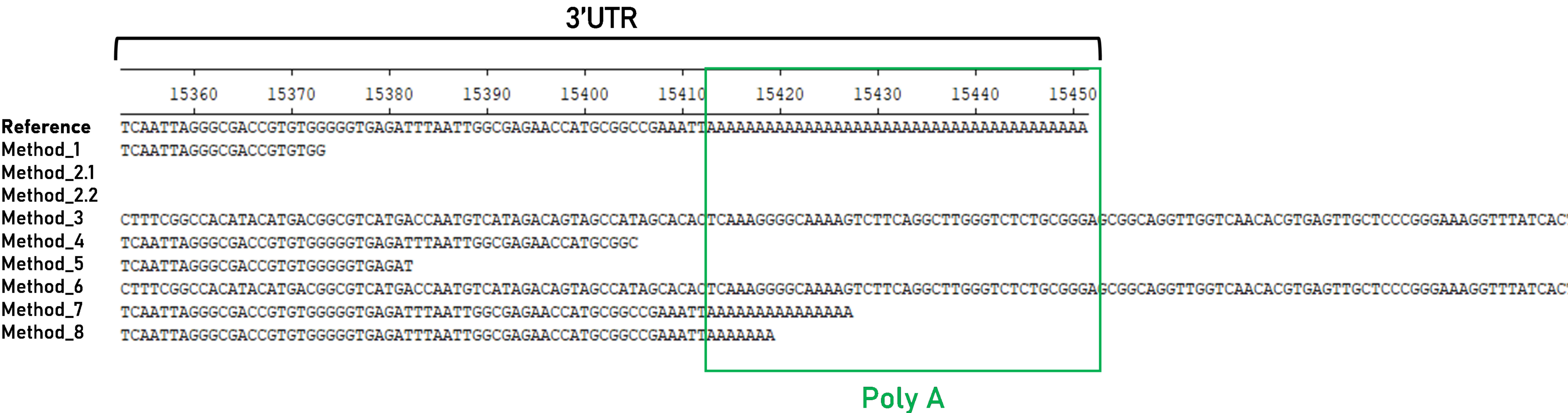

Supplement: Supplementary file 6 — Additional file 6. BLAST results of newly assembled PRRSV whole-genome sequences. [file 12985_2022_1790_MOESM6_ESM.pdf]

## Hypervariable Region

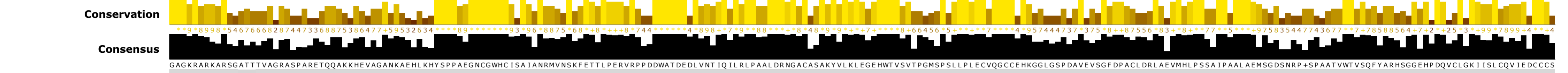

### Hypervariable Region II

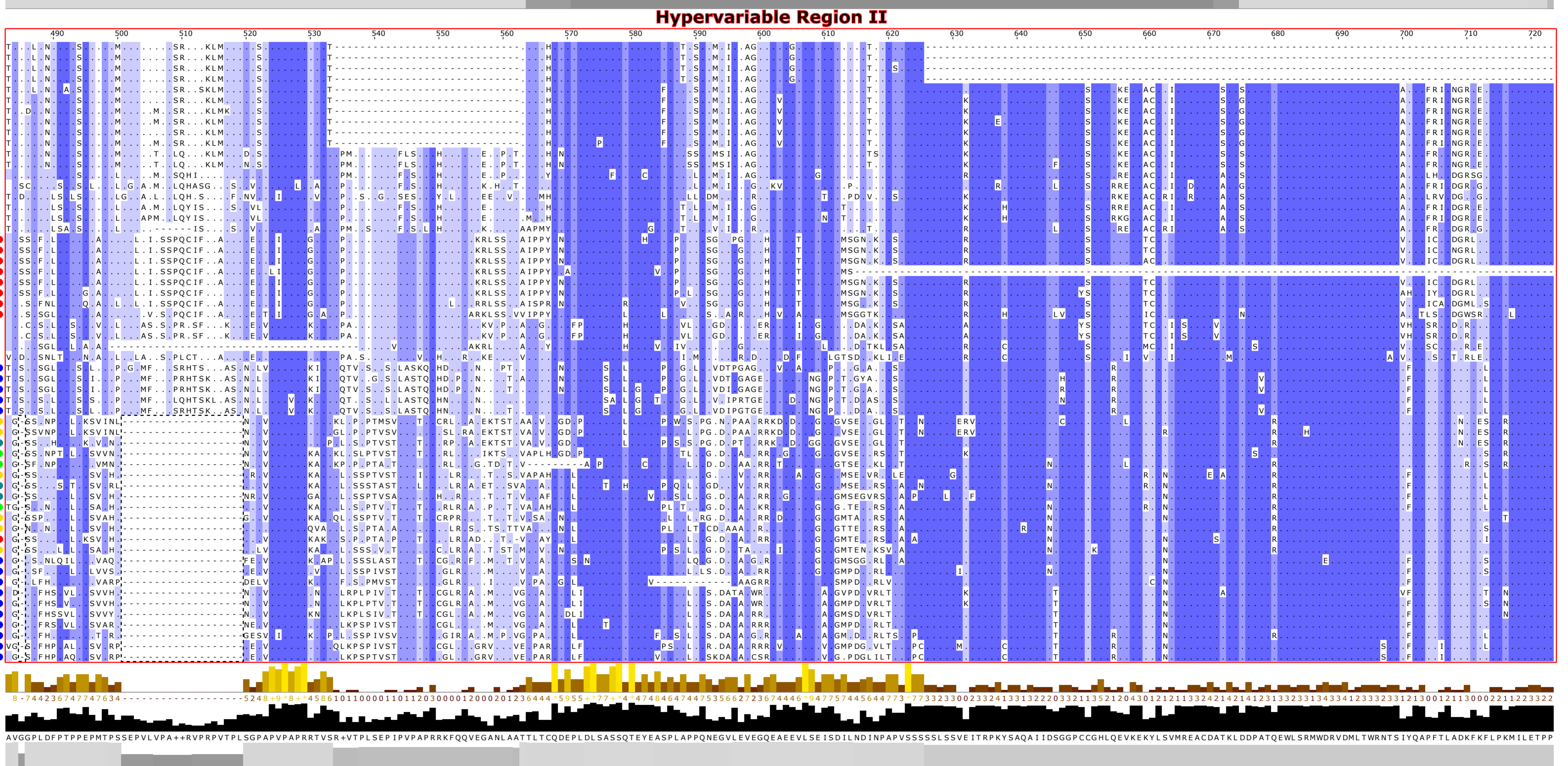

## Hypervariable Region II

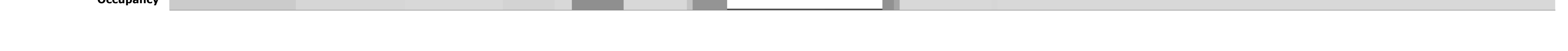

Supplement: Supplementary file 9 — Additional file 9. SimPlot analysis querying potential major parent strains against Korean PRRSV isolates. [file 12985_2022_1790_MOESM9_ESM.pdf]
