# Supplementary material for: Whole-genome sequencing and genetic characteristics of representative porcine reproductive and respiratory syndrome virus (PRRSV) isolates in Korea
Source: Virol J. 2022 Apr 11;19:66. doi: 10.1186/s12985-022-01790-6 (PMC8996673; doi:10.1186/s12985-022-01790-6)
Supplement: Supplementary file 8 — Additional file 8. Multiple sequence alignment of PRRSV2 NSP2, with the “111 + 1 + 19” deletions indicated with black dashed-line boxes. [file 12985_2022_1790_MOESM8_ESM.pdf]

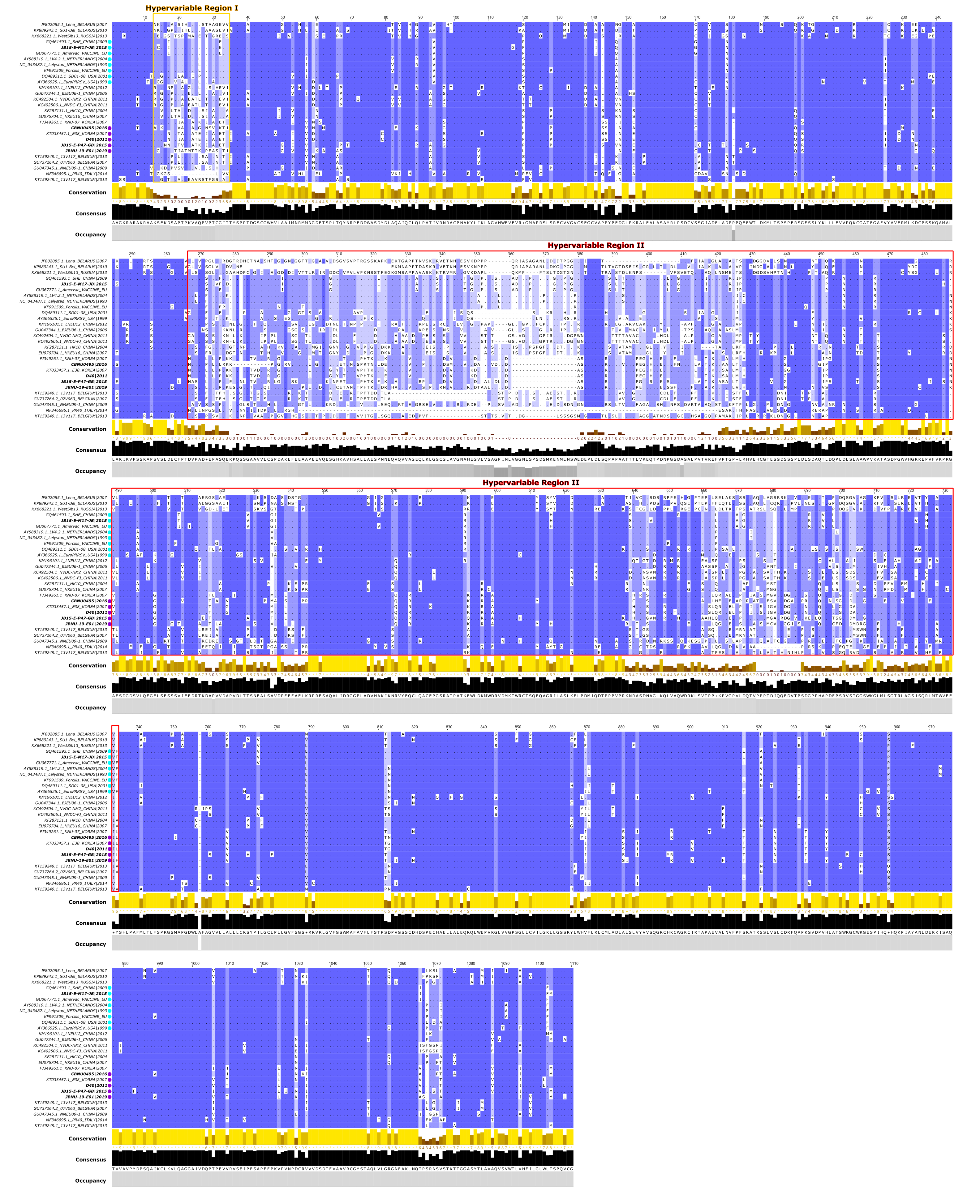
Hypervariable Region I
Hypervariable Region II
Hypervariable Region II
JF802085.1_Lena_BELARUS|2007
KP889243.1_SU1-Bel_BELARUS|2010
KX668221.1_WestSib13_RUSSIA|2013
GQ461593.1_SHE_CHINA|2009
JB15-E-M17-JB|2015
GU067771.1_Amervac_VACCINE_EU
AY588319.1_LV4.2.1_NETHERLANDS|2004
NC_043487.1_Lelystad_NETHERLANDS|1993
KF991509_Porcilis_VACCINE_EU
DQ489311.1_SD01-08_USA|2001
AY366525.1_EuroPRRSV_USA|1999
KM196101.1_LNEU12_CHINA|2012
GU047344.1_BJEU06-1_CHINA|2006
KC492504.1_NVDC-NM2_CHINA|2011
KC492506.1_NVDC-FJ_CHINA|2011
KF287131.1_HK10_CHINA|2004
EU076704.1_HKEU16_CHINA|2007
FJ349261.1_KNU-07_KOREA|2007
CBNU0495|2016
KT033457.1_E38_KOREA|2007
D40|2011
JB15-E-P47-GB|2015
JBNU-19-E01|2019
KT159249.1_13V117_BELGIUM|2013
GU737264.2_07V063_BELGIUM|2007
GU047345.1_NMEU09-1_CHINA|2009
MF346695.1_PR40_ITALY|2014
KT159249.1_13V117_BELGIUM|2013
Conservation
Consensus
Occupancy
