# Supplementary material for: Whole-genome sequencing and genetic characteristics of representative porcine reproductive and respiratory syndrome virus (PRRSV) isolates in Korea
Source: Virol J. 2022 Apr 11;19:66. doi: 10.1186/s12985-022-01790-6 (PMC8996673; doi:10.1186/s12985-022-01790-6)

PRRSV1 (EU) strains

Amervac vs Kor PRRSV1

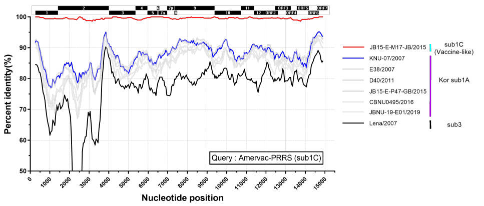

KNU-07 vs Kor PRRSV1

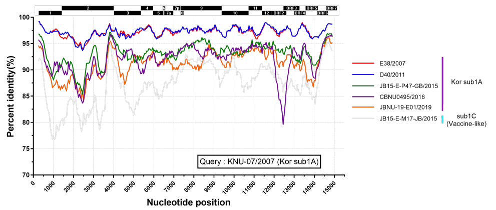

CBNU0495 vs Kor PRRSV1

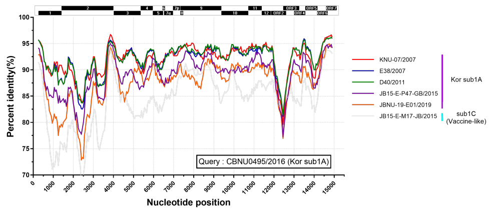

PRRSV2 (NA) strains

MLV (L5) vs Kor PRRSV2 L5

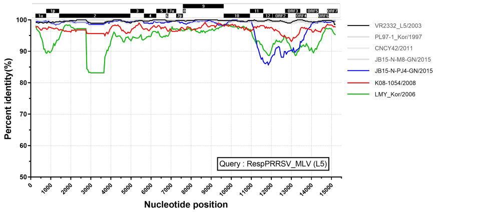

MLV (L5) vs Kor PRRSV2

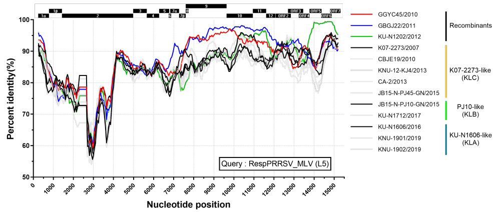

NADC30(L1) vs Kor PRRSV2

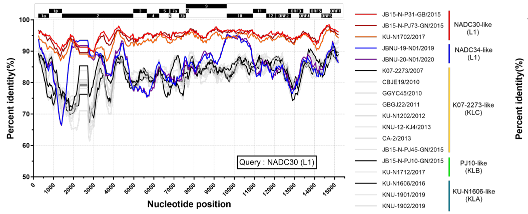

NADC34(L1) vs Kor PRRSV2

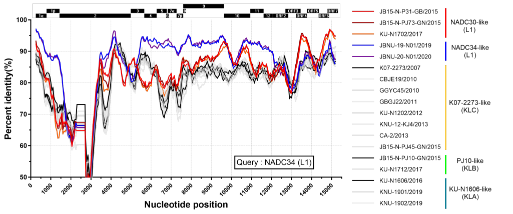

K07-2273(KLC) vs Kor PRRSV2

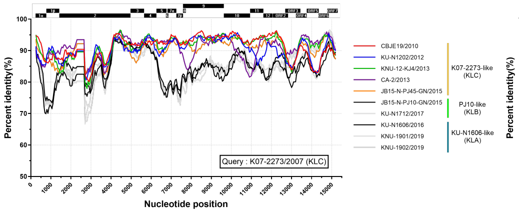

2015-N-PJ10-GN(KLB) vs Kor PRRSV2

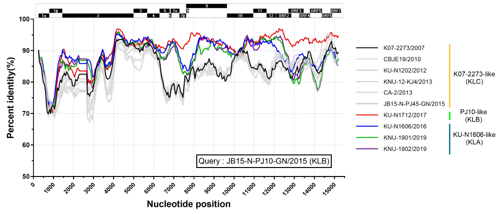

KU-N1606(KLA) vs Kor PRRSV2

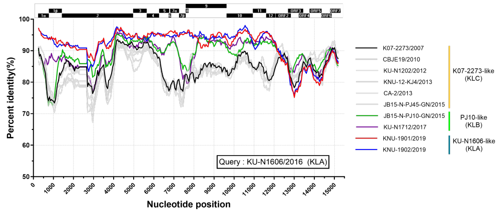

Supplement: Supplementary file 10 — Additional file 10. P value of seven methods (RDP, GENECONV, BootScan, Maxchi, Chimaera, SiScan, and 3Seq) applied in RDP4 software. [file 12985_2022_1790_MOESM10_ESM.pdf]
